# Dietary patterns and diabetes mellitus among people living with and without HIV: a cross-sectional study in Tanzania

**DOI:** 10.3389/fnut.2023.1105254

**Published:** 2023-05-17

**Authors:** Evangelista Malindisa, Haruna Dika, Andrea M. Rehman, Mette Frahm Olsen, Filbert Francis, Henrik Friis, Daniel Faurholt-Jepsen, Suzanne Filteau, George PrayGod

**Affiliations:** ^1^Department of Physiology, The Catholic University of Health and Allied Sciences Bugando, Mwanza, Tanzania; ^2^Mwanza Research Centre, National Institute for Medical Research, Mwanza, Tanzania; ^3^Faculty of Epidemiology and Population Health, London School of Hygiene & Tropical Medicine, London, United Kingdom; ^4^Department of Infectious Diseases, Rigshospitalet, Copenhagen, Denmark; ^5^Tanga Research Centre, National Institute for Medical Research, Tanga, Tanzania; ^6^Department of Nutrition, Exercise, and Sports, University of Copenhagen, Copenhagen, Denmark

**Keywords:** dietary patterns, associated factors, prediabetes, diabetes, HIV

## Abstract

**Background:**

Due to the complexity of human diets, it is difficult to relate single foods to health outcomes. We aimed to identify the dietary patterns and associated factors and to assess the association of dietary patterns with prediabetes/diabetes among adults living with and without HIV in Tanzania.

**Methods:**

Diet data were collected by a food frequency questionnaire (FFQ) and dietary patterns were derived by principal component analysis (PCA) and reduced rank regression (RRR). The associations between dietary patterns and associated factors as well as with prediabetes/diabetes were assessed using multinomial logistic regression and presented by marginal plots.

**Results:**

Of 572 recruited, 63% were people living with HIV. The mean (±SD) age was 42.6 (±11.7) years and 60% were females. The PCA identified two major dietary patterns, i.e., vegetable-rich pattern (VRP) and vegetable-poor pattern (VPP) whereas RRR identified one dietary pattern, i.e., carbohydrate-dense pattern (CDP). In comparison to females, males had higher adherence to VPP and CDP, but less to VRP. Higher socioeconomic status was associated with higher adherence to VRP and VPP but low adherence to CDP. Compared to HIV-negative participants, people living with HIV had higher adherence to VRP but less adherence to CDP. Compared to younger people, older people had lower adherence to VPP. High adherence to CDP or VRP was positively associated with prediabetes. Higher adherence to VRP was associated with a borderline decrease in diabetes. No association was observed between VPP with either prediabetes or diabetes.

**Conclusion:**

Our findings suggest that dietary patterns may impact the risk of prediabetes and diabetes differently. Awareness of the health benefits of VRP should be encouraged in the community, especially for men who seem to consume fewer vegetables. Longitudinal studies are needed to explore the contribution of dietary patterns to prediabetes/diabetes development in sub-Saharan Africa.

## Background

Many studies have found that the intake of individual macronutrients and micronutrients may influence health (1, 2). However, due to the complexity of the human diet, it is difficult to conclude if a single food, ingredient, or nutrient is associated with health outcomes (3). Therefore the focus has shifted to investigating the role of the entire diet on health outcomes (3).

Several factors such as food availability, commercial interests, and socioeconomic status (SES) may influence food choices and eating behaviors (4). Studies in high-income countries have suggested higher SES is associated with having a healthier dietary pattern (5). However, similar studies in sub-Saharan Africa (SSA) are few and inconsistent. For example in Ghana, school children from high SES households had an unhealthy eating pattern (6), while in Seychelles wealthy families had a high intake of fruits and vegetables (4). Infectious diseases such as HIV and tuberculosis could influence the choice of dietary patterns (7). In South Africa, it was found that dietary patterns differed by HIV status, but this has not been widely replicated in other parts of Africa (8). A recent study in El Salvador found that children living with HIV had unhealthy dietary patterns, and suggested that dietary patterns in people living with HIV might contribute to malnutrition-related diseases in this population (9). However, such data are limited for both people living with HIV and people with no HIV in SSA. This hinders the development of strategies to improve the intake of healthier diets to reduce the risk of non-communicable diseases (NCDs) like diabetes.

The interplay of traditional risk factors such as obesity, energy-dense diets, and physical inactivity is implicated in reduced insulin secretion, increased insulin resistance, and the development of diabetes mellitus (10). However, diabetes in SSA may have a different etiology, as suggested by data showing a high prevalence of diabetes in non-overweight people and low insulin secretion as well as insulin resistance (11, 12). We aimed to determine dietary patterns and factors associated with and explore the association between dietary patterns with prediabetes and diabetes among adults in Mwanza, Tanzania.

## Methods

### Study design and area

This analysis was part of the role of environmental enteropathy on HIV-associated diabetes (REEHAD) study, a cross-sectional study investigating the links between environmental enteric dysfunction and diabetes in Mwanza (clinicaltrials.gov NCT03713502). REEHAD is nested within the Chronic Infections, Co-morbidities, and Diabetes in Africa (CICADA) study, a prospective cohort study investigating the burden of, and risk factors for diabetes and other NCDs among Tanzanian adults with and without HIV (13). The REEHAD study enrolled participants between May 2019 and March 2021.

### Sample size estimation and recruitment

The sample size was calculated using Open Source Epidemiologic Statistics for Public Health (OpenEpi) sample size calculator for cross-sectional studies 2013 (14, 15). Based on prior data from the CICADA study, we assumed that the proportion of diabetes measured by oral glucose tolerance test (OGTT) in people adhering to healthy patterns and unhealthy patterns are 3 and 10%, respectively (13). To demonstrate these differences between the highest and lowest terciles with 80% power at a 5% significance level, we required a minimum sample size of 555, divided into terciles of adherence to dietary patterns. Participants were consecutively recruited during their clinic visits until this sample size was reached.

### Data collection

#### Socio-demographic characteristics and NCDs behavioral risk factors

Data on socio-demographic characteristics including age, sex, employment status, marital status, and education level were collected using pre-tested structured questionnaires. In addition, data on possession of assets (residential house, electric or gas cooker, bicycle, motorcycle, car, sewing machine, radio, television, air-conditioner, mobile phone, animals (goats/cows), chickens, boat, and any rented property), source of water for domestic use and type of toilet used were collected and used to compute SES score using principal component analysis (PCA) (16) from which the first component was grouped in terciles (i.e., lower, middle and upper) for analysis. The World Health Organization (WHO) Global Physical Activity Questionnaire (GPAQ) was used to collect reported data on the level of physical activity (17). Total physical activity was computed to metabolic equivalents of tasks (MET) in minutes per week and categorized as an adequate level of physical activity if MET was ≥600 as recommended by WHO (18). Smoking status was elicited and grouped as never smoked, past smoker (quit smoking for >1 year), and current smoker (smoking within the past 1 year). Alcohol consumption was grouped as never consumed, past consumption (quit intake for >1 year), and current consumption (consuming within the past 1 year). HIV tests were done during CICADA recruitment as published (13). Antiretroviral therapy (ART) history was collected from participants’ ART cards and verified with ART clinic records.

#### Anthropometric measurements

Body weight was determined to the nearest 0.1 kg using a digital scale (Seca, Germany) while participants were barefoot and with minimal clothing. Height was measured to the nearest 0.1 cm using a stadiometer fixed to the clinic wall (Seca, Germany). BMI was categorized as underweight (BMI < 18.5 kg/m^2^), normal weight (BMI 18.5–24.9 kg/m^2^), or overweight/obese (BMI ≥ 25 kg/m^2^). Waist circumference was measured by a non–stretchable measuring tape to the nearest 0.1 cm taken at the midpoint between the lower costal margin and the iliac crest, with the subject standing erect in a relaxed position and feet placed 25–30 cm apart. Values >94 cm for males and > 80 cm for females were regarded as abdominal obesity according to WHO (19).

#### Diabetes assessment

Diabetes was defined by 2 h OGTT. Participants were contacted 1 day before the clinic visit and instructed to fast overnight for at least 8 h and come to the clinic early in the morning (between 8 am to 10 am). Blood for glucose measurement (Hemocue 201 RT, Hemocue AB, Angelholm, Sweden) was drawn from fasted participants. They were then given 82.5 g of dextrose monohydrate (equivalent to 75 g of anhydrous glucose) diluted in 250 ml of drinking water to drink within 5 min for OGTT. We used WHO criteria to classify participants with normal blood glucose (<7.8 mmol/L), prediabetes (7.8–11.0 mmol/L), or diabetes (≥11.1 mmol/L). In addition, we looked at HbA1c as a supplementary test, because it is recommended by WHO in diabetes diagnosis (20, 21), however it underestimates diabetes in hemoglobinopathy and 20% of people in the study population have sickle cell trait (22, 23). Participants were classified as normal (≤ 5.6%), prediabetes (5.7–6.4%), and diabetes (≥6.5%).

#### Dietary assessment

A food frequency questionnaire (FFQ) was used to assess dietary habits. The FFQ was adapted from the validated Africa/Harvard School of Public Health Partnership for Cohort Research &Training (PaCT) questionnaire (24) which was modified to fit the Tanzanian context. The modified FFQ had over 350 food items that are found in the Tanzania food composition tables (25). Participants were asked to recall the usual intake of food items in terms of frequency and quantity for the past 12 months, and these were then aggregated into 30 food groups based on their nutrient profile and culinary use (26). The groups included refined grains, unrefined grains, mixed dish grains, natural fruits and juices, artificially sweetened beverages, legumes, red meat (beef, goat, and lamb), chicken-based dishes, fish-based dishes, milk, eggs, pork, banana dishes, potato dishes, chips and crisps, yams, pumpkins, cassava dishes, green vegetables, cruciferous vegetables, dark-yellow-orange vegetables, tomatoes, alcohol, sweets and desserts, edible insects, honey and sugar, coffee, mixed-dish tubers, tea without sugar and seeds and nuts.

#### Generation of dietary patterns

Based on the reported frequencies of intake of each food group, participants were assigned to the eating frequency of each food group into “never” if they never ate, “rarely” if they ate 1–3 times a month, “moderate” if they ate once a week, “regular” if they ate 2–6 per week and “very regular” if they ate on daily basis. Dietary patterns were derived using PCA and reduced rank regression (RRR) as described by Hoffman et al. (27). The two methods are conceptually different, but complementary in understanding the role of dietary patterns on health outcomes (28). PCA derives dietary patterns through data reduction; however, literature has shown that PCA-derived patterns might not be associated with health outcomes of interest (27). On the other hand, RRR analysis derives dietary patterns using intermediate variables or response variables that are known to be associated with the outcome of interest (27, 29). RRR requires a minimum of 2 response variables and in the current analysis, the two response variables were selected based on their strength of association with diabetes and based on previous studies, BMI and waist circumference (30, 31) were selected; both variables were left-skewed and were log-transformed before analysis. For PCA analysis, we checked sample adequacy using Kaiser-Meyer-Olken (KMO) test (32). We also rotated component loadings of the retained factors to create less correlation between the components and facilitate interpretability (32). A Scree plot was used to retain the components for subsequent analysis (Supplementary Figure 1); factors above the elbow of the scree plot were retained (32).

### Data management and analysis

Data were entered into CSPro and analyzed in STATA 15. Background characteristics of the study participants (i.e., age categories, sex, marital status, SES, employment status, alcohol drinking, physical activity, smoking, body fat, BMI, HIV status, and education level) were presented as counts (percentages) or means (SD) as appropriate. In addition, we investigated the differences in continuous and categorical variables between REEHAD participants included in this analysis and those not included using student *t*-test and chi-squared test, respectively. For the analysis of factors associated with dietary patterns, dietary pattern scores (defined by PCA or RRR and divided into terciles, upper tercile representing high adherence to the pattern and the lower tercile representing low consumption) were the primary outcomes while background factors (age, sex, HIV status, SES, employment status, education level, and physical activity) were the main predictor factors. For analyses exploring associations of dietary patterns and diabetes, dietary patterns were the exposure while the above background factors were adjusted for potential confounders. Multinomial logistic regression was used to assess associations between outcome variables and independent variables. In addition, the associations between dietary patterns with prediabetes and diabetes were presented by marginal effects plots. *p* value <0.05 was considered statistically significant.

## Results

**Figure 1:**
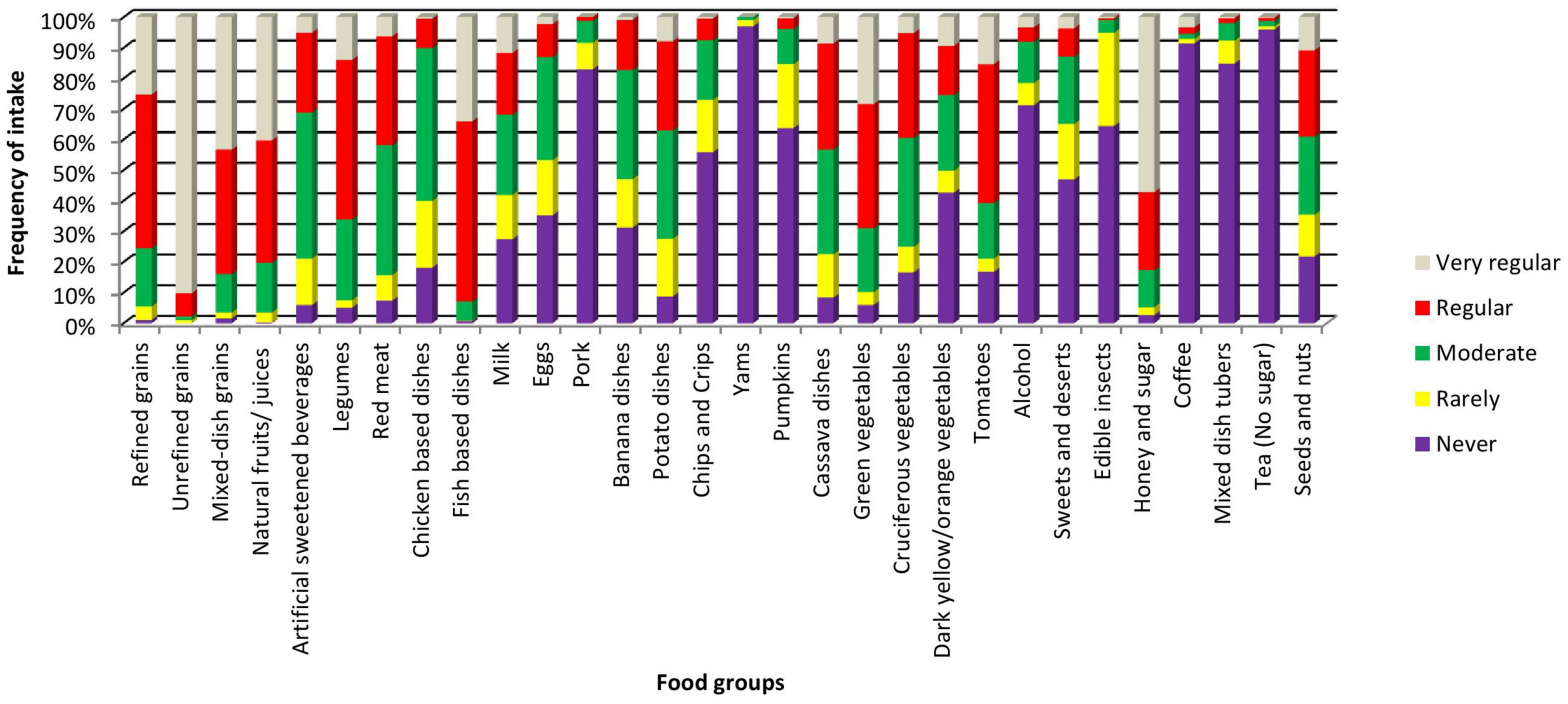
Frequencies of intake of the food groups. Very regular; if eaten on daily basis, regular; if eaten 2-6 per week, moderate; if eaten once a week, rarely; if eaten1-3 times a month, never; if was never eaten.

Of 1,174 participants recruited in the REEHAD study, 572 (49%) with diet data were included in the current analysis. There were no differences between REEHAD participants included and those not included in the analysis except that those included had more self-employed participants (overall *p* = 0.01; Table 1). The mean (±SD) age of the study participants was 43 (±12) years and 59% were females. Over half (57%) of participants were married/cohabiting and most (79%) were self-employed. About 6% were current smokers, 24% were current alcohol drinkers and 63% were people living with HIV and on ART. Prediabetes and diabetes were present in 25 and 4%, respectively (Table 1). The intake frequency of all food groups has been presented in Figure 1.

**Table 1:** Characteristics of the role of environmental enteropathy on HIV-associated diabetes (REEHAD) participants included in the analysis (*N* = 572) versus those not analyzed (*N* = 600).

Characteristic	Categories	Participants analyzed^1^	Participants not analyzed^1^	Overall *p*-value
Age, mean (SD)^2^	-	42.6 ± 11.7	39.8 ± 11.6	0.91
Sex	Female sex	338 (59)	355 (59.2)	0.95
Education	Never attended school	107 (19)	97 (16.2)	0.08
	Primary school	387 (67.7)	394 (65.7)	
	Secondary School and Higher	78 (13.6)	109 (18.2)	
Marital status	Married/cohabiting	324 (56.7)	336 (56.0)	0.3
	Widowed	86 (15.0)	77 (12.8)	
	Separated/divorced	139 (24.3)	149 (24.8)	
	Single/never married	23 (4.0)	38 (6.3)	
Employment	Salaried employee	60 (10.5)	89 (14.8)	**0.01**
	Self employed	451 (78.8)	425 (70.8)	
	Unemployed/House wife	61 (10.7)	86 (14.4)	
SES terciles	Lower	210 (36.8)	200 (33.3)	0.2
	Middle	198 (34.7)	200 (33.3)	
	Upper	163 (28.6)	200 (33.3)	
Smoking status	Never	433 (75.7)	456 (76.0)	0.9
	Past-smoker	104 (18.2)	105 (17.5)	
	Current smoker	35 (6.1)	39 (6.5)	
Alcohol drinking	Never	174 (30.4)	185 (30.8)	0.9
	Past-drinker	260 (45.5)	266 (44.4)	
	Current-drinker	138 (24.1)	149 (24.8)	
Physical activity	Active (≥600 MET minutes/week)	547 (95.5)	556 (92.7)	0.05
Waist circumference	Abnormal	255 (44.50)	280 (46.67)	0.46
BMI (kg/m^2^), mean (SD)^2^	-	22.5 (4.4)	23.4 (4.9)	0.97
HIV status	HIV positive	362 (63.3)	365 (60.8)	0.38

1 Values are *n* (%) unless otherwise specified.

2 Standard deviations.

^1^BMI calculated as weight (kg)/height (m); ^2^MET, Metabolic equivalent of tasks; ^3^Diabetes was defined by WHO guidelines using oral glucose test results (33).The bold values show the statistical significant values.

### Dietary patterns

In the PCA of 30 food groups, the first two components, with eigenvalues of 4.3 and 1.7, explained 20% of the total variation and were retained for further analysis (32) (Supplementary Table 1). Kaiser-Meyer-Olkin’s measure was 0.8, indicating sampling adequacy (32). Food groups with factor loadings ≥|0.25| were used to name the patterns in each component (26), and high negative loadings were rarely seen (Supplementary Table 2). Component 1 had high positive loadings for natural fruits and juices, bananas, potatoes, green vegetables, cruciferous vegetables, dark orange vegetables, and tomatoes; therefore this component was named vegetable-rich pattern (VRP). Component 2 had high positive loadings for artificially sweetened beverages, red meat, milk, chips and crisps, and alcohol, and a high negative loading for tomatoes, this was named the vegetable-poor pattern (VPP). RRR analysis yielded two factors, of which the first factor explained much more (98%) of the total variance in the intermediate response variables than the second; the first factor was therefore retained for the subsequent analysis. The total variation of the response variables explained by RRR factors was 17.1%, while the variation of predictors, i.e., the food groups, explained by RRR factors was 8.2%. Supplementary Table 3 shows the factor loadings for the RRR retained factors. Refined grains had a factor loading of |0.72| while the rest of the food groups had factor loadings less than |0.25|, this pattern has therefore been named carbohydrate-dense pattern (CDP).

### Factors associated with dietary patterns

In fully adjusted models (Table 2), males had lower adherence to VRP than women (OR 0.4, 95% CI 0.3, 0.7). In comparison to participants in the lower socio-economic class, those in the upper socio-economic class had higher adherence to VRP (OR 2.3, 95% CI 1.3, 4.1). Compared with those who never attended school; primary school and secondary school (or above) attendees had higher adherence to VRP (OR 1.8, 95% CI 1.0, 3.3 and OR 6.8, 95% CI 2.6, 17.6 respectively). Participants with HIV had higher adherence to VRP than those without HIV (OR 2.0, 95% CI 1.3, 3.2). Age, physical activity, and employment status had no significant association with VRP (Table 2).

**Table 2:** Socio-demographic characteristics associated with terciles of vegetable-rich pattern scores.

		Univariable model				Multivariable model			
		Middle tercile		Upper tercile		Middle tercile		Upper tercile	
		Relative risk ratio (95% CI)	Overall *P*	Relative risk ratio, 95% CI	Overall *P*	Adjusted relative risk ratio, 95% CI	Overall *P*	Adjusted relative risk ratio, 95% CI	Overall *P*
Sex	Female	Reference	0.09	Reference	**0.01**	Reference	**0.01**	Reference	**<0.001**
	Male	0.7 (0.5, 1.1)		0.6 (0.4, 0.9)		0.5 (0.3, 0.8)		0.4 (0.3, 0.7)	
Age (years)	18–30	Reference	0.4	Reference	0.2	Reference	0.5	Reference	0.8
	31–40	1(0.5, 1.9)		0.8 (0.4, 1.5)		1.3 (0.7, 2.5)		1.1 (0.6, 2.1)	
	41–50	0.7 (0.4, 1.3)		0.6 (0.3, 1.1)		0.9 (0.5, 1.8)		0.8 (0.4, 1.6)	
	50+	0.8 (0.4, 1.5)		0.5 (0.3, 1)		1.3 (0.6, 2.6)		0.96 (0.5, 1.97)	
SES^1^	Lower	Reference	**0.003**	Reference	**<0.001**	Reference	**0.02**	Reference	**0.02**
	Middle	1.7 (1.1, 2.8)		1.5 (0.9, 2.5)		1.8 (1.1, 2.9)		1.5 (0.9, 2.5)	
	Upper	2.5(1.5, 4.2)		2.8 (1.6, 4.6)		2.1 (1.2, 3.8)		2.3(1.3, 4.1)	
PA^2^	Not active	Reference	0.9	Reference	0.8	Reference	0.95	Reference	0.98
	Active	1 (0.4, 2.6)		1.1 (0.4, 2.98)		1.03 (0.4, 2.9)		0.98 (0.3, 2.8)	
Edu. level^3^	Never attended	Reference	**0.002**	Reference	**<0.001**	Reference	**0.01**	Reference	**<0.001**
	Primary	1.2 (0.7, 1.96)		1.8 (1, 3)		1.1 (0.7, 1.96)		1.8 (1.03, 3.3)	
	Secondary and above	4.2 (1.8, 9.7)		6.6(2.8, 15.6)		4.0 (1.6, 10.1)		6.8 (2.6, 17.6)	
Employment	Unemployed/house wife	Reference	0.09	Reference	0.6	Reference	0.07	Reference	0.6
	Self-employment/other	1.4(0.7, 2.7)		1 (0.5, 1.9)		1.6 (0.8, 3.4)		1.2 (0.6, 2.4)	
	Salary employed	2.7 (1.1, 6.6)		1.5 (0.6, 3.7)		3.2 (1.2, 8.7)		1.7 (0.6, 4.5)	
HIV status	Negative	Reference	0.09	Reference	**0.04**	Reference	**0.01**	Reference	**0.003**
	Positive	1.4 (0.95, 2.2)		1.6 (1, 2.4)		1.8 (1.1, 2.8)		2(1.3, 3.2)	

1 Socioeconomic status.

2 Physical activity.

3 Education level.

Variables (i.e., sex, age, HIV, socioeconomic status, physical activity, education level and employment) adjusted for each other. Lower tercile is the reference for the middle and upper terciles.

The bold values show the statistical significant values.

Compared to women, males had higher adherence to VPP (OR 3.9, 95% CI 2.3, 6.3). In comparison to younger age groups; older age groups had lower adherence to VPP (OR 0.3, 95% CI 0.2, 0.7). Participants in the middle and upper SES class had higher adherence to VPP than participants in the lower class (OR 2.3, 95% CI 1.4, 4.0 and OR 5.4, 95% CI 2.9, 10.2 respectively). Selfand salary-employed participants had higher adherence to VPP than the unemployed and housewives (OR 2.7, 95% CI 1.1, 6.3 and OR 4.5, 95% CI 1.5, 13.8 respectively). Physical activity, education level, and HIV status had no significant association with VPP scores (Table 3).

**Table 3:** Socio-demographic characteristics associated with terciles of vegetable-poor pattern scores.

		Univariable model				Multivariable model			
		Middle tercile		Upper tercile		Middle tercile		Upper tercile	
		Relative risk ratio, (95% CI)	Overall *P*	Relative risk ratio, 95% CI	Overall *P*	Adjusted Relative risk ratio, 95% CI	Overall *P*	Adjusted Relative risk ratio, 95% CI	Overall *P*
Sex	Female	Reference	**0.02**	Reference	**<0.001**	Reference	**0.01**	Reference	**<0.001**
	Male	1.7 (1.1, 2.6)		3.9 (2.5, 5.9)		1.8 (1.1, 3)		3.9 (2.3, 6.3)	
Age (years)	18–30	Reference	0.2	Reference	0.06	Reference	0.1	Reference	**0.01**
	31–40	0.7 (0.4, 1.3)		0.7 (0.4, 1.3)		0.7 (0.3, 1.3)		0.6 (0.3, 1.2)	
	41–50	0.5 (0.3, 0.9)		0.5 (0.2, 0.9)		0.5 (0.2, 0.9)		0.3 (0.2, 0.7)	
	50+	0.8 (0.4, 1.5)		0.5 (0.3, 0.98)		0.7 (0.3, 1.4)		0.3 (0.2, 0.7)	
SES^1^	Lower	Reference	**0.01**	Reference	**<0.001**	Reference	**0.01**	Reference	**<0.001**
	Middle	1.2 (0.8, 1.9)		2.2 (1.4, 3.7)		1.3 (0.8, 2)		2.3 (1.4, 4)	
	Upper	2.4 (1.4, 4.2)		6.2 (3.6, 10.9)		2.4 (1.3, 4.4)		5.4 (2.9, 10.2)	
PA^2^	Not active	Reference	0.99	Reference	0.07	Reference	0.8	Reference	0.4
	Active	0.99 (0.3, 3.1)		0.4 (0.2, 1.1)		1.2 (0.4, 4)		0.6 (0.2, 1.9)	
Edu. level^3^	Never attended	Reference	**0.02**	Reference	**<0.001**	Reference	0.2	Reference	0.30
	Primary	1.9 (1.2, 3.2)		2.4 (1.4, 4.2)		1.6 (0.9, 2.7)		1.5 (0.8, 2.7)	
	Secondary and above	2.6 (1.1, 5.8)		7.4 (3.4, 16.2)		1.4 (0.6, 3.4)		2 (0.8, 4.9)	
Employment	Unemployed/house wife	Reference	0.9	Reference	**<0.001**	Reference	0.9	Reference	**0.03**
	Self-employment/other	1.1 (0.6, 1.9)		2.8 (1.3, 6.2)		1.1 (0.6, 2)		2.7 (1.1, 6.3)	
	Salary employed	1.3 (0.5, 3.4)		9.8 (3.6, 27)		0.9 (0.3, 2.5)		4.5 (1.5, 13.8)	
HIV status	Negative	Reference	0.8	Reference	0.8	Reference	0.2	Reference	0.1
	Positive	1.1 (0.7, 1.6)		0.9 (0.6, 1.4)		1.4 (0.9, 2.2)		1.5 (0.9, 2.4)	

1 Socioeconomic status.

2 Physical activity.

3 Education level.

Variables (i.e., sex, age, HIV, socioeconomic status, physical activity, education level and employment) adjusted for each other. Lower tercile is the reference for the middle and upper terciles.

The bold values show the statistical significant values.

In comparison to women, males had higher adherence to CDP (OR 1.7, 95% CI 1.1, 2.8). Participants in the upper socioeconomic class had lower adherence to CDP compared to those in the low socioeconomic class (OR 0.3, 95% CI 0.2, 0.6). Participants living with HIV had lower adherence to CDP than participants living without HIV (OR 0.5, 95% CI 0.31, 0.8). Age, physical activity, education level, and employment status were not associated with CDP (Table 4).

**Table 4:** Socio-demographic characteristics associated with terciles of carbohydrate-dense pattern scores.

		Univariable model				Multivariable model			
		Middle tercile		Upper tercile		Middle tercile		Upper tercile	
		Relative risk ratio, (95% CI)	Overall *P*	Relative risk ratio, 95% CI	Overall *P*	Adjusted Relative risk ratio, 95% CI	Overall *P*	Adjusted Relative risk ratio, 95% CI	Overall *P*
Sex	Female	Reference	**0. 003**	Reference	**<0.001**	Reference	**<0.001**	Reference	**0.03**
	Male	0.5 (0.4, 0.8)		0.4 (0.2, 0.6)		3.8 (2.3, 6.3)		1.7 (1.1, 2.8)	
Age (years)	18–30	Reference	0.2	Reference	0.05	Reference	0.5	Reference	0.5
	31–40	1.4 (0.8, 2.8)		0.8 (0.4, 1.5)		0.9 (0.5, 1.9)		1.6(0.9, 3)	
	41–50	0.8 (0.4, 1.6)		0.5 (0.3, 0.9)		1.5 (0.7, 2.9)		1.6 (0.8, 3)	
	50+	0.9 (0.5, 1.8)		0.6 (0.3, 1.1)		0.98 (0.5, 2.1)		1.2 (0.6, 2.5)	
SES^1^	Lower	Reference	**<0.001**	Reference	**<0.001**	Reference	**<0.001**	Reference	**0.001**
	Middle	0.5 (0.3, 0.8)		0.6 (0.4, 1.1)		0.5 (0.3, 0.8)		0.6 (0.3, 1)	
	Upper	0.1 (0.06, 0.2)		0.4 (0.2, 0.6)		0.1 (0.04, 0.2)		0.3 (0.2, 0.6)	
PA^2^	Not active	Reference	0.5	Reference	0.3	Reference	0.5	Reference	0.9
	Active	1.4 (0.6, 3.6)		1.6 (0.6, 4.2)		0.7 (0.2, 2)		1.1 (0.4, 3.2)	
Edu. level^3^	Never attended	Reference	0.4	Reference	**0.01**	Reference	0.2	Reference	0.5
	Primary	1.3 (0.8, 2.2)		1.7 (1, 3)		0.6 (0.3, 1.1)		0.8 (0.5, 1.5)	
	Secondary and above	1.6 (0.8, 3.5)		3.5 (1.6, 7.3)		0.5 (0.2, 1.2)		0.6 (0.3, 1.4)	
Employment	Unemployed/house wife	Reference	0.9	Reference	0.2	Reference	0.6	Reference	0.7
	Self-employment/other	1.04 (0.5, 2.1)		0.7 (0.4, 1.4)		1.1 (0.5, 2.3)		1.1 (0.6, 2.3)	
	Salary employed	1.2 (0.5, 3.02)		1.3 (0.5, 3.1)		0.7 (0.2, 2)		0.8 (0.3, 2.2)	
HIV status	Negative	Reference	0.2	Reference	0.4	Reference	**0.02**	Reference	**0.003**
	Positive	0.8 (0.5, 1.2)		1.2 (0.8, 1.9)		0.5 (0.3,0.9)		0.5 (0.31, 0.8)	

1 Socioeconomic status.

2 Physical activity.

3 Education level.

Variables (i.e., sex, age, HIV,SES, physical activity, education level and employment) adjusted for each other.

Lower tercile is the reference for the middle and upper terciles. The bold values show the statistical significant values.

### Association of dietary patterns with prediabetes and diabetes

**Figure 2:**
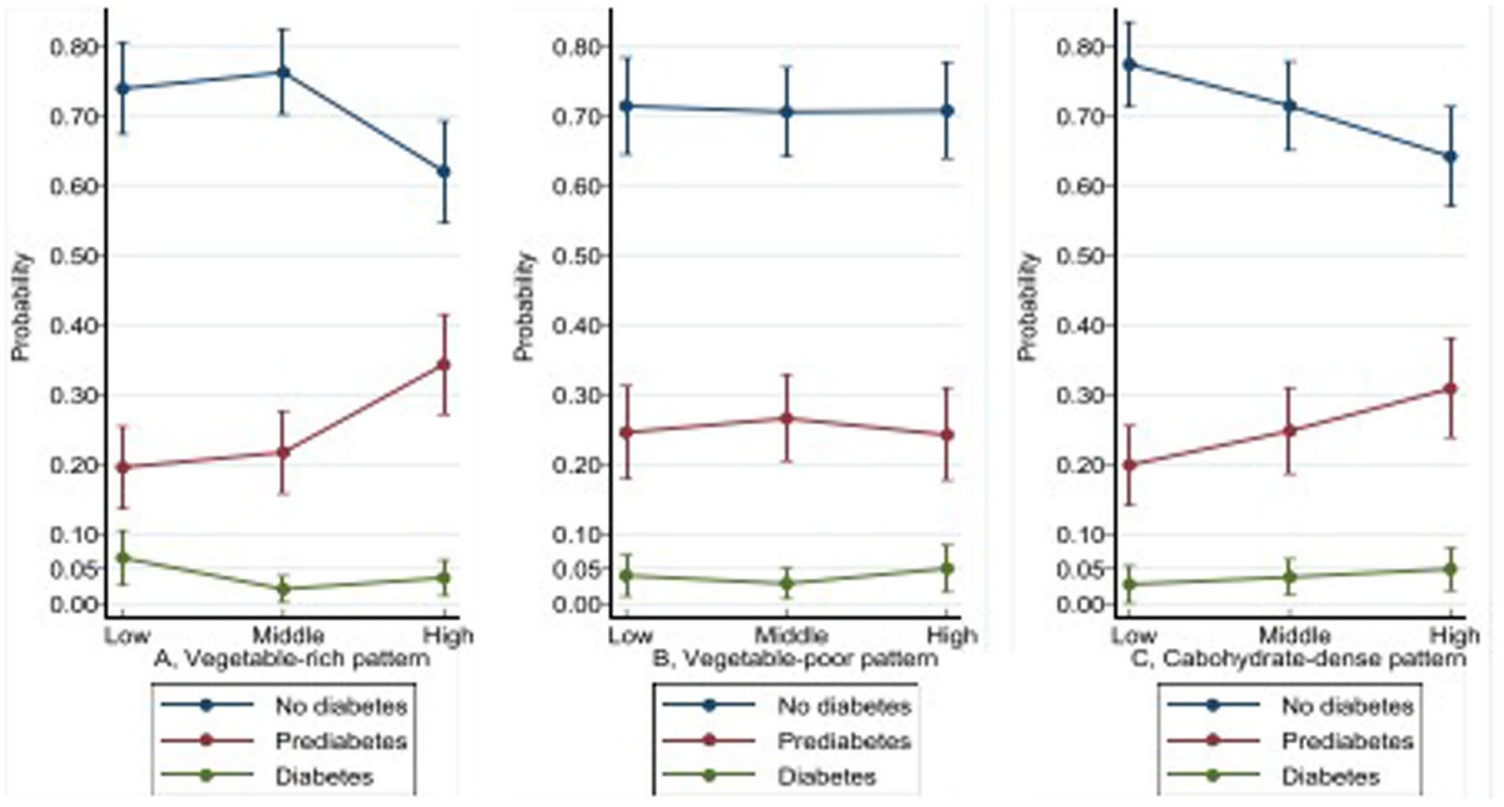
Distribution of the probabilities of prediabetes and diabetes across terciles of dietary patterns Vegetable-rich pattern: (vegetables, banana based dishes, potato based dishes, natural fruits and juices). Vegetable–poor: (artificial sweetened beverages, milk, red meat, alcohol, chips and crisps). Carbohydrate-dense pattern: (unrefined grains (rice, millet, wheat, maize)). Multivariable analyses have been adjusted for the age, sex, socio-economic status, education level, physical activity, HIV status and for other pattern in PCA-derived patterns. Diabetes status was based on blood glucose at 120 min in an oral glucose tolerance test: no-diabetes <7.8 mmol/L (reference); prediabetes: 7.8–11.0 mmol/L; diabetes ≥11.1 mmol/L.

Using 2 h OGTT data, in a fully adjusted models, compared to the lower tercile, high VRP consumption was associated with a greater risk of prediabetes (ARRR = 2.12, 95% CI: 1.24, 3.66) but inversely associated with diabetes (ARRR = 0.26, 95% CI: 0.07, 0.95). High adherence to CDP was positively associated with prediabetes (ARRR = 1.92, 95% CI 1.12, 3.29), but there was no association with frank diabetes. VPP consumption was not significantly associated with either prediabetes or diabetes. Figure 2 shows the marginal plots for the probabilities of prediabetes and diabetes across the terciles of dietary patterns after fully adjusted multinomial logistic regression models. In VRP, the probability of having prediabetes increases in the upper tercile, while there is no change in the probability of having diabetes. There are no observed changes in the probability of having prediabetes or diabetes in the upper tercile of VPP. In CDP, there is an increased probability of having prediabetes, and a slight increase in the probability of diabetes in the upper tercile.

Using HbA1c data, in fully adjusted models, we observed no significant associations between prediabetes and diabetes with terciles of all 3 dietary patterns (Supplementary Figure 2).

## Discussion

Among adult Tanzanians, we found that higher adherence to VRP was positively associated with the female sex, higher SES, high level of education, and living with HIV. Higher adherence to VPP was positively associated with male sex, higher SES, younger age, and having employment. Higher adherence to CDP was positively associated with the male sex, lower SES, and living without HIV. Higher adherence to VRP was associated with a lower probability of having diabetes, but surprisingly it was associated with a higher probability of pre-diabetes. Higher adherence to CDP was associated with increased probabilities of prediabetes as well as diabetes. VPP was not associated with either prediabetes or diabetes.

Studies on the association between SES and dietary patterns in high-income countries have observed people from higher socioeconomic classes eat healthier foods, including vegetables and fruits, than those in the low socioeconomic class (4, 5, 34, 35). Data on SES and dietary patterns in low-and middle-income countries (LMIC) are few with different directions of associations (5). A multinational study conducted among children in 11 LMICs including Kenya (the ISCOLE study) observed that higher SES was associated with a healthy diet (35). On the other hand, a review of cross-sectional studies of LMIC found a positive association between higher SES and unhealthy diets in some studies (5). The finding that SES is associated with higher scores of both healthy (VRP) and unhealthy (VPP) diets suggests that relatively wealthy people in Tanzania consume both healthy and unhealthy foods. Unlike the situation in high-resource settings where healthy foods including vegetables and fruits are expensive, in Tanzania vegetables are less expensive compared to fast foods (36). Thus, the intake of unhealthy foods among those from high socioeconomic classes may be due to inappropriate perceptions, cultural beliefs, and lack of education on the role of vegetables and fruits in healthy living, although we found the association was independent of the level of education. On the other hand, low adherence to VRP among those with low SES may reflect the inability to afford healthy foods due to extreme poverty.

Similar to many other studies, gender differences have an impact on adherence to dietary patterns; this is due to differences in roles, behavior, and attitudes that exist between different genders. Compared to women, men had higher scores of VPP and CDP. Women had higher scores of VRP but relatively lower scores of VPP and CDP. This result is in agreement with many other studies (37, 38), and the possible explanation is that women may avoid foods that may increase their weight, although there is no evidence for this in Tanzania where cultural perspectives of weight gain have been different (37). Another explanation would be that men go more frequently to fast food restaurants; studies in the USA have reported that men eat more restaurant meals and fast foods than women (39, 40). This is likely to be the same in Tanzania, although published data are limited, where it is rare to find vegetables and fruits incorporated in the food menu of the local restaurants, possibly explaining why men do not take as much of VRP. The majority of those who drink alcohol are men, and most bars sell beers together with red meat (Swahili “nyamachoma”) and fried potatoes with eggs (Swahili “chips mayai”) (41).

The positive association between HIV infection and higher scores of VRP observed in this study may be explained by the fact that national guidelines on support for people with HIV emphasize that people with HIV should be encouraged to meet their vitamin and mineral needs from their diet by eating a variety of fruits and vegetables (42).

In the current study, increasing age (>40 years) was negatively associated with higher scores of VPP. Similar findings have been reported in Taiwan (43), the Republic of Seychelles (4), and Serbia where older people ate healthier diets than the young populations (44). This may suggest that people over 40 years of age may be aware of their increased risk of NCDs like diabetes, therefore they may be avoiding unhealthy diets. It is also possible that the dietary transition from traditional healthy diets to junk foods has less affected older people who have preserved their preference more for VRP and less for VPP than the younger ones.

We expected to observe a negative association between VRP with prediabetes and diabetes (45), unexpectedly; the VRP was associated with an increased risk of prediabetes. We also expected positive associations between VPP and CDP with prediabetes and diabetes (46). We found that the CDP was associated with a higher risk of prediabetes but, VPP showed no association with either prediabetes or diabetes. Similar to our study, a recent study in Tanzania reported a lack of association between unhealthy dietary pattern with adiposity in adolescents (47). Another study in Tanzania has found a lack of association between being overweight and prediabetes/diabetes (13), indicating that prediabetes/diabetes in SSA may not be linked to being overweight and hence not driven by the traditional risk factors known from studies in high-income countries.

Our findings could be explained by the fact people with high SES take both VRP and VPP. The intake of unhealthy diets may overwhelm the beneficial effects of VRP leading to a higher risk of prediabetes. Although we adjusted for SES, based on an asset index, this may not have completely removed the effect of SES which includes other aspects in addition to asset ownership. Unlike associations of prediabetes with presumably healthy diets, we found that VRP lowered diabetes risk. This may reflect the beneficial effects of vegetables and fruits intake since it is known that vegetable and fruits contain antioxidants that may reduce insulin resistance and β-cells apoptosis thus reducing the risk of diabetes (48). While recall bias is a known problem with diet assessment by FFQ, this was likely to be similar across diabetes groups since we did not specifically inform study participants that FFQ were meant to collect data to assess their risk for diabetes, and data collectors were unaware of diabetes status of participants.

This study had several strengths and limitations. First, we included both people living with HIV and people with no HIV, which increases the generalizability of findings. Secondly, we have used two different methods to derive dietary patterns; while PCA-derived patterns reflect general dietary behavior in the population, RRR-derived pattern reflects a dietary pattern that associates with the response variables (BMI and waist circumference) which are the markers of the diseases of interest (prediabetes and diabetes in the current study) (29). The use of the two methods has led to different patterns that have fostered the understanding of the behavioral dietary patterns but also the patterns that associate with the markers of diabetes (49). This study also had several limitations: it was cross-sectional thus causality cannot be confirmed and involved participants from previous cohorts so survival bias may have been introduced. Another limitation is, these participants were from a cohort, it is possible that some of them were previously diagnosed with prediabetes or diabetes and could have changed their diet recently. Also, we have used 2 h OGTT results to define prediabetes and diabetes in the current study; this may underestimate the diabetes burden in this population. Although we also used HbA1c, this is less reliable in this population, it under-diagnoses diabetes in hemoglobinopathies (20), and about 20% of this population has sickle cell trait (22, 23). Fasting blood glucose levels have been high in this population (13), questioning the influence of other factors as reported elsewhere (13, 50).

## Conclusion

Our findings suggest that dietary patterns may impact the risk of prediabetes and diabetes differently. Education for adherence to VRP should be encouraged in the community, especially for men who seem to consume fewer vegetables. Longitudinal studies should be conducted to explore the contribution of dietary patterns on prediabetes and diabetes mellitus in sub-Saharan Africa.

## Data availability statement

The datasets presented in this article are not readily available because include de-identified data that cannot be shared publicly, and according to Tanzanian ethics guidelines, it is not possible to share any data including de-identified data without approval of the Medical Research Coordinating Committee (MRCC). Data are available from the National Institute for Medical Research (NIMR) and can be shared with researchers who meet the criteria for access to confidential data only after completing a data transfer agreement and approval by the MRCC. Requests to access the datasets should be directed to ethics@nimr.or.tz.

## Ethics statement

This study received ethical approval from the Medical Research Coordinating Committee of the National Institute for Medical Research and the joint Catholic University of Health and Allied Sciences/Bugando Medical Center Research Ethics and Review Committee. In addition, the CICADA study was approved by the Research Ethics Committee of the London School of Hygiene and Tropical Medicine, and consultative approval was provided by the National Committee on Health Research Ethics in Denmark. All methods were under the Declaration of Helsinki. Participants were enrolled after written informed consent and those with diabetes and other illnesses were referred to Sekou-Toure referral hospital for care.

## Author contributions

EM, DF-J, AR, HF, SF, and GP conceived the study. EM, HD, and GP collected data. EM analyzed data with help from FF and AR and wrote the first draft. All authors contributed to the article and approved the submitted version.

## Funding

This project is part of the EDCTP2 program supported by the European Union (grant agreement number: TMA2017GSF-1965-REEHAD). It received additional support from the Ministry of Foreign Affairs of Denmark through DANIDA Fellowship Centre (grant: 16-P01-TAN). The funding agencies had no role in the study design, data collection and analysis, and decision to publish results. The information contained in this publication is not the responsibility of the funding agencies, and any use of it is not their responsibility either.

## Conflict of interest

The authors declare that the research was conducted in the absence of any commercial or financial relationships that could be construed as a potential conflict of interest.

## Publisher’s note

All claims expressed in this article are solely those of the authors and do not necessarily represent those of their affiliated organizations, or those of the publisher, the editors and the reviewers. Any product that may be evaluated in this article, or claim that may be made by its manufacturer, is not guaranteed or endorsed by the publisher.

## Supplementary material

SUPPLEMENTARY FIGURE 1 Sree plot of Eigenvalues after PCA.

SUPPLEMENTARY FIGURE 2 Distribution of the probabilities of prediabetes and diabetes across terciles of dietary patterns Vegetable-rich pattern: (vegetables, banana based dishes, potato based dishes, natural fruits and juices).Vegetable–poor: (artificial sweetened beverages, milk, red meat, alcohol, chips and crisps). Carbohydrate-dense pattern: (unrefined grains (rice, millet, wheat, maize)). Multivariable analyses have been adjusted for the age, sex, socio-economic status, education level, physical activity, HIV status and for other pattern in PCA-derived patterns. Diabetes status was based on HbA1c: no-diabetes ≤ 5.6% (reference); prediabetes: 5.7-6.4% diabetes ≥ 6.5%.

## Abbreviations

FFQ: Food frequency questionnaire
BMI: Body mass index
WHO: World Health Organization
GPAQ: Global physical activity questionnaire
HIV: Human immunodeficiency virus
SSA: Sub Saharan Africa
NCD: Non-communicable diseases
PCA: principal component analysis
RRR: reduced rank regression
MET: The metabolic equivalent of tasks
REEHAD: Role of environmental enteropathy on HIV associated diabetes
CICADA: Chronic infections, co-morbidities, and diabetes in Africa cohort.
VRP: Vegetable-rich pattern
VPP: Vegetable-poor dietary pattern
CDP: Carbohydrate-dense dietary pattern
ARRR: adjusted relative risk ratio
