# Supplementary material for: Dietary patterns and diabetes mellitus among people living with and without HIV: a cross-sectional study in Tanzania
Source: Front Nutr. 2023 May 17;10:1105254. doi: 10.3389/fnut.2023.1105254 (PMC10230058; doi:10.3389/fnut.2023.1105254)
Supplement: Supplementary file 1 [file Table_1.DOCX]

| **Supplementary Table 1:** Eigen values and variance of predictor variables explained by each PCA components | | | |
| --- | --- | --- | --- |
| Component number | Eigen values | Proportion of explained variance | Cumulative proportion of explained variance |
| Comp1 | 4.32533 | 0.1442 | 0.1442 |
| Comp2 | 1.7212 | 0.0574 | 0.2016 |
| Comp3 | 1.61113 | 0.0537 | 0.2553 |
| Comp4 | 1.31895 | 0.0440 | 0.2992 |
| Comp5 | 1.23435 | 0.0411 | 0.3404 |
| Comp6 | 1.19753 | 0.0399 | 0.3803 |
| Comp7 | 1.15667 | 0.0386 | 0.4188 |
| Comp8 | 1.08877 | 0.0363 | 0.4551 |
| Comp9 | 1.03095 | 0.0344 | 0.4895 |
| Comp10 | .994866 | 0.0332 | 0.5227 |
| Comp11 | .98723 | 0.0329 | 0.5556 |
| Comp12 | .942508 | 0.0314 | 0.5870 |
| Comp13 | .89685 | 0.0299 | 0.6169 |
| Comp14 | .882304 | 0.0294 | 0.6463 |
| Comp15 | .864464 | 0.0288 | 0.6751 |
| Comp16 | .860302 | 0.0287 | 0.7038 |
| Comp17 | .825215 | 0.0275 | 0.7313 |
| Comp18 | .794462 | 0.0265 | 0.7578 |
| Comp19 | .750972 | 0.0250 | 0.7828 |
| Comp20 | .71909 | 0.0240 | 0.8068 |
| Comp21 | .688186 | 0.0229 | 0.8297 |
| Comp22 | .672281 | 0.0224 | 0.8521 |
| Comp23 | .638013 | 0.0213 | 0.8734 |
| Comp24 | .62721 | 0.0209 | 0.8943 |
| Comp25 | .610182 | 0.0203 | 0.9146 |
| Comp26 | .584645 | 0.0195 | 0.9341 |
| Comp27 | .542693 | 0.0181 | 0.9522 |
| Comp28 | .497062 | 0.0166 | 0.9688 |
| Comp29 | .476668 | 0.0159 | 0.9847 |
| Comp30 | .459918 | 0.0153 | 1.0000 |
