# Supplementary figures and images for: Dietary patterns and diabetes mellitus among people living with and without HIV: a cross-sectional study in Tanzania
Source: Front Nutr. 2023 May 17;10:1105254. doi: 10.3389/fnut.2023.1105254 (PMC10230058; doi:10.3389/fnut.2023.1105254)

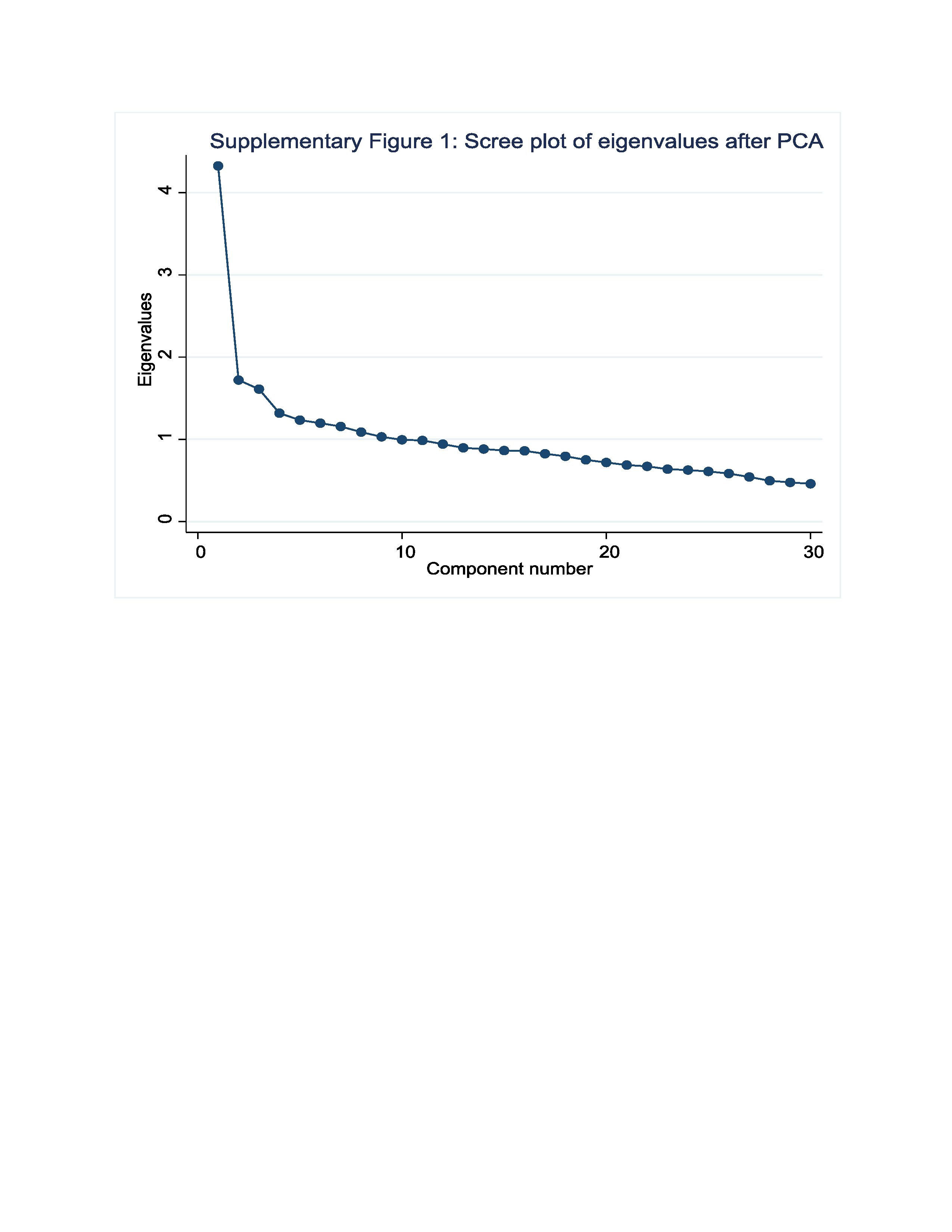

Supplement: SUPPLEMENTARY FIGURE 1 — Sree plot of Eigenvalues after PCA. [file Image_1.JPEG]

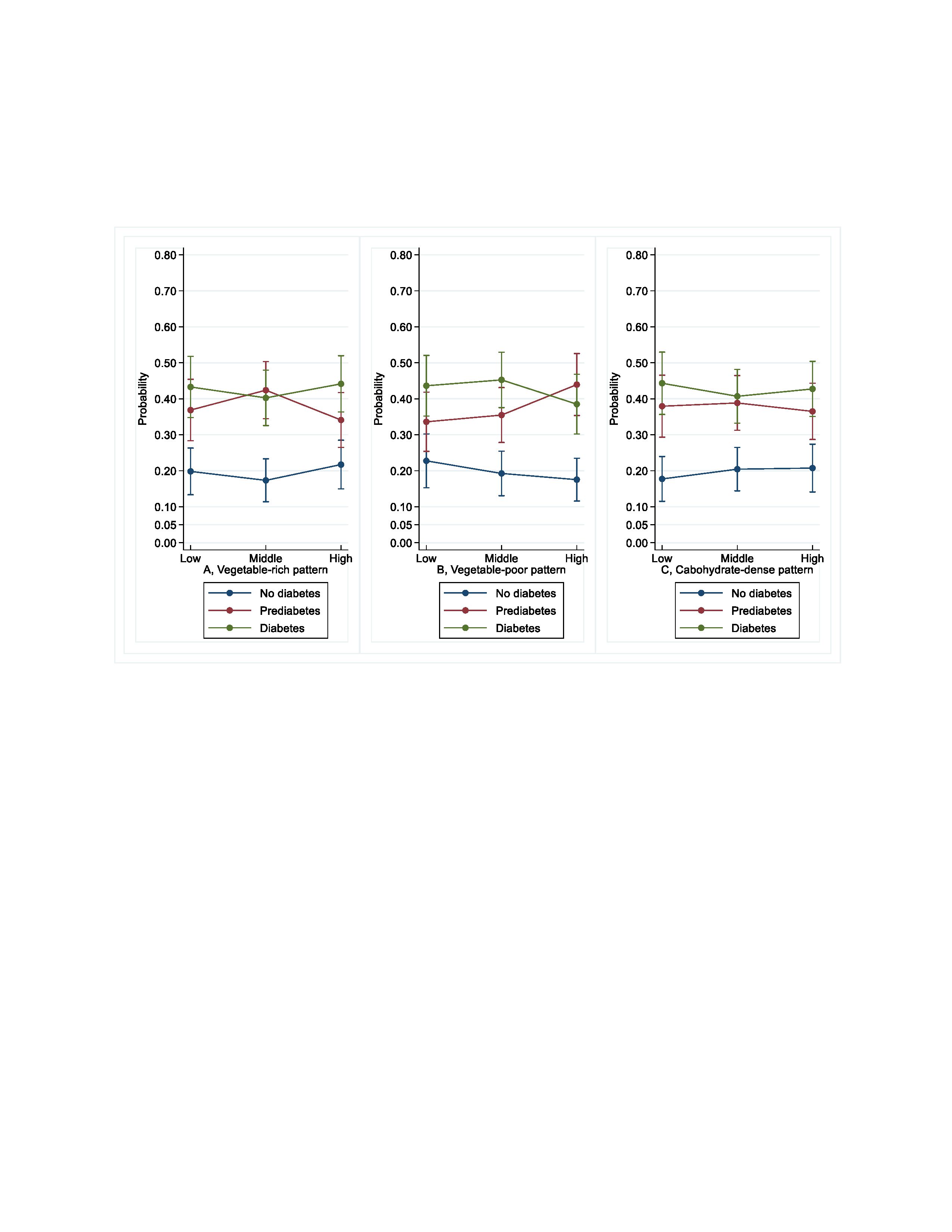

Supplement: SUPPLEMENTARY FIGURE 2 — Distribution of the probabilities of prediabetes and diabetes across terciles of dietary patterns Vegetable-rich pattern: (vegetables, banana based dishes, potato based dishes, natural fruits and juices).Vegetable–poor: (artificial sweetened beverages, milk, red meat, alcohol, chips and crisps). Carbohydrate-dense pattern: (unrefined grains (rice, millet, wheat, maize)). Multivariable analyses have been adjusted for the age, sex, socio-economic status, education level, physical activity, HIV status and for other pattern in PCA-derived patterns. Diabetes status was based on HbA1c: no-diabetes ≤ 5.6% (reference); prediabetes: 5.7-6.4% diabetes ≥ 6.5%. [file Image_2.JPEG]
